# Substrate Pre-loading Influences Initial Colonization of GAC Biofilter Biofilms

**DOI:** 10.3389/fmicb.2020.596156

**Published:** 2021-01-12

**Authors:** Wen Qin, Frederik Hammes

**Affiliations:** ^1^School of Civil and Transportation Engineering, Guangdong University of Technology, Guangzhou, China; ^2^Department of Environmental Microbiology, Eawag-Swiss Federal Institute of Aquatic Science and Technology, Dübendorf, Switzerland

**Keywords:** biofiltration, biological activated carbon (BAC), granular activated carbon (GAC), carbon source loading, colonization, microbial resource management (MRM), stability

## Abstract

Microbial community composition and stability affect pollutant removal for biological/granular activated carbon (BAC/GAC) processes. Here, we pre-loaded the organic carbon substrates sucrose, lactose, and Lysogeny Broth (LB) medium onto new GAC prior to use and then tested whether this substrate pre-loading promoted development of biofilms with high coverage that remained stable for prolonged operational periods. Temporal dynamics of the biomass and microbial community on the GAC were monitored *via* flow cytometry (FCM) and sequencing, respectively, in both batch and continuous-flow experiments. In comparison with the non-loaded GAC (control), the initial biofilm biomass on substrate-loaded GAC was 3–114 times higher, but the initial richness was considerably lower (only accounting for 13–28% of the control). The initial community compositions were significantly different between batch and continuous-flow column experiments, even when loaded with the same substrates. In the continuous-flow column experiments, both biomass and microbial community composition became remarkably similar to the control filters after 64 days of operation. From these findings, we conclude that substrate-loaded GAC could enhance initial colonization, affecting both biomass and microbial community composition. However, the biomass and composition did not remain stable during long-term operation due to continuous dispersal and competition from influent bacteria.

## Introduction

Biological/granular activated carbon (BAC/GAC) filtration, which combines adsorption with biodegradation, is a promising method for the removal of organics, is general, and specifically removes micro-pollutants from drinking water and wastewater (Kirisits et al., 2001; Kalkan et al., 2011; Chu et al., 2012; Jantarakasem et al., 2020; Zhiteneva et al., 2020). Biodegradation plays an important role in pollutant removal in BAC filters over prolonged operation (Liang and Chiang, 2007; Velten et al., 2011; Gibert et al., 2013). Therefore, management of the microbial community composition on the GAC particles in the filter is essential for enhancing removal efficiency of targeted pollutants. However, new biofilters typically require a long start-up period (2–3 months) to reach a steady state in biofilm concentration, community composition, and targeted pollutant effluent concentration (Velten et al., 2011; Han et al., 2013; Zheng et al., 2018). Furthermore, it is conceivable that targeted pollutants could not be removed efficiently due to failed/insufficient colonization of the specific species required for their degradation. Thus, targeted initial GAC colonization, enrichment of specific communities, and maintenance of the stability of these enriched bacteria on GAC filters are essential to shorten the start-up time and to enhance removal efficiency of targeted pollutants.

At present, the methods for enhancing pollutant removal efficiency by accelerating biofilm formation in the BAC filters include the artificial inoculation of specific functional bacteria (seed bacteria; Gao et al., 2010; Zhang et al., 2013) and the use of spent GAC as filter media (Sun et al., 2018). These two methods are Microbial Resource Management (MRM) strategies, which have been proposed as a concept to solve practical problems through using microorganisms (i.e., obtaining and maintaining an efficient microbial community; Verstraete et al., 2007; Read et al., 2011; Vlaeminck et al., 2012). Seed bacteria could realize the functionalization of the initial biofilm on the activated carbon carrier and efficiently remove the target pollutants according to the water quality characteristics (Qin et al., 2016). However, some studies reported difficulties in maintaining long-term stability because of the low diversity and richness of the seed bacteria (the number of synthetic community species <20) could not resist the continuous competition from indigenous bacteria in influent (Zhang et al., 2013; Mallon et al., 2015). A previous study reported that the spent GAC, collected from a carbon contactor that had been in service for about 3 years, enabled biodegradation of N-nitrosodimethylamine (NDMA) and 22 Pharmaceuticals and Personal Care Products (PPCPs; Sun et al., 2018). The above studies confirmed that quickly realizing the functionalization of the biofilm was an effective strategy to improve the pollutant removal efficiency of the BAC/GAC process. In the present study, we approached the problem from a different perspective, with the view manipulating the initial community through manipulating the available nutrients on the GAC.

In a GAC filter, initial biofilm formation starts with the colonization of the new activated carbon carrier (blank niche) by bacteria in the filter influent. This is followed by a change of biofilm composition during long-term operation as a consequence of microbial community succession through continuous competition between bacteria present in the influent and the initial colonizing community. Four fundamental processes (i.e., dispersal, selection, drift, and diversification; (Vellend, 2010) shaping community dynamics determine the success of such competition (Kinnunen et al., 2016). Dispersal in an operational filter is continuous (i.e., bacteria constantly flow into the system). Diversification, the development of new species, and drift, stochastic changes through demographic processes of “birth” (or division) and death (Hubbell, 2001; McGill, 2003), in a filter are difficult to regulate artificially. Selection is likely a key determinant of initial colonization and the only factor that is possible to control. The impact of selection on competition depends on the resident community’s composition and the environmental conditions (van Nevel et al., 2013; Mallon et al., 2015). Therefore, carbon as an essential nutrient for microorganisms is a possible key to simultaneously regulate the rapid functionalization and stabilization of biofilms in the GAC start-up process. In addition, previous studies demonstrated the strategy that biofunctionalization of materials using an optimal carbon source to form a biofilm as a live, protective barrier for prolonged protection against pathogenic colonization worked well (Lopez et al., 2011; Trautner et al., 2012; Zhu et al., 2015). Hence, we argue that rapid initial bacterial colonization driven by the presence of a specific carbon source would result in rapid, high biofilm coverage on the carrier material, which might limit dispersal, adhesion, and competition from the filter influent community.

In the present study, we suggest a MRM strategy to alter the initial microbial community through altering the initial nutrient conditions on the GAC surface. The concept is to create a favorable GAC surface by pre-loading with specific biodegradable compounds and then select and enrich specific microbial communities from the targeted water during initial colonization. The ideal outcome would be the formation of a high-coverage biofilm on the carbon-loaded GAC particles at the beginning of operation. Moreover, a preferred outcome would be the maintenance of a stable community with prolonged resistance against competition from bacteria in the filter influent. The aim of this paper was to test the basic premises of this strategy. To this end, we chose several common substrates as proof-of-concept carbon sources, with different characteristics that should arguably select for different bacterial communities, and adsorbed those substrates on GAC particles. Subsequently, we exposed the different types of pre-loaded GAC to groundwater in both batch and continuous-flow column experiments. We monitored the temporal dynamics of biomass and microbial community composition on the GAC to investigate (1) whether GAC loaded with different substrates selects a specific microbial community and (2) whether the community remains stable during operation.

## Materials and Methods

### Adsorption of Different Substrates on GAC

#### Cleaning and Sterilization of GAC

New GAC [bituminous coal-based type, FILTRASORB 400, Chemviron Carbon (European Operations of Calgon Carbon Corporation, United States)] was washed with nanopure water and incubated at 80°C for 6 h for drying and at 100°C for 2 h for pasteurization prior to use. Clean GAC was transferred into sterile Schott bottles for storage. The surface area (BET N_2_ method) was 1,050 m^2^/g.

#### Substrate Selection

We selected five well-known organic-carbon substrates with varied bio-availability [glucose, acetate, sucrose, lactose, and Lysogeny Broth (LB) medium] for the study. Glucose and sucrose can be used by most kinds of bacteria through the glycolytic pathway. Acetate, as the precursor of acetyl coenzyme in the tricarboxylic acid cycle, also can be utilized by the majority of bacteria. Lactose can be used only by bacteria which have the lactase enzyme. LB medium is a common medium for culturing heterotrophic bacteria; it is a complex carbon source, and contains organic-nitrogen (i.e., carbon bound) and other nutrients (Tryptone, Yeast extract, and inorganic salt). Ten grams of dry weight (DW) GAC were added into 1 L flasks containing 500 ml of 100 mg-C/L sterile substrate solution in triplicate for each substrate, where mg-C/L refers to the dissolved organic carbon (DOC) concentration of each substrate. The flasks were incubated with 150 rpm shaking for 24 h at room temperature. DOC was analyzed before the addition of GAC and after 24 h shaking to assess adsorption. Subsequently, the GAC was added into new 1 L flasks containing 500 ml sterile nanopure water. To assess desorption, DOC was analyzed before the addition of GAC and after 24 h shaking. The amount of carbon adsorbed/desorbed by GAC (X_carbon_) was calculated with the formula as followed, where DOC_0h_ represents the initial DOC, DOC_24h_ represents the DOC after 24 h shaking, V represents the volume of the substrate solution, M_GAC_ represents the DW of GAC.

(1) $$X_{carbon}=\frac{\left(DOC_{0h}-DOC_{24h}\right)\times V}{M_{GAC}}$$

#### Preparation of Substrate-Loaded GAC

For batch experiments, the substrate-loaded GAC was prepared as described as above. For continuous-flow column experiments: substrate-loaded GAC was described as above, except that initial amounts were increased. 250 g DW GAC were added into 2 L flasks containing 1 L of 2.5 g-C/L sterile substrate solution, and all other steps were as above.

### Water Quality of the Inoculum (Groundwater)

The inoculum used in both batch and continuous-flow column experiments was untreated groundwater (Dübendorf, Switzerland). The groundwater had consistent water quality and suspended bacterial concentrations. The details of water quality of the groundwater are shown in Table 1.

**Table 1:** Water quality and suspended bacterial concentrations of groundwater.

Ammonium (μg N/L)	TN (mg N/L)	TP (μg P/L)	TOC (mg C/L)	DOC (mg C/L)	TCC (×10^5^ cells/ml)	ICC (×10^5^ cells/ml)	pH
13.5	5.2	38	1.9	1.3	3.5 ± 0.8	2.1 ± 0.7	7.64

TN is total nitrogen, TP is total phosphorous, TOC is total organic carbon, DOC is dissolved organic carbon, TCC is total cell concentration, and ICC is intact cell concentration.

### Batch Experiments

#### Groundwater as the Inoculum and Water Matrix

Ten grams of DW GAC loaded with sucrose, lactose, and LB medium were added into separate flasks containing 500 ml groundwater. Ten grams of DW new GAC was used as a control. Subsequently, the flasks were incubated for 5 days with 150 rpm shaking at room temperature. Experiments were done in triplicate. Water samples were collected during 5 days shaking to analyze bacterial concentrations. GAC samples were collected on Day 5 for detection of biofilm biomass and microbial communities. The schematic overview of the batch experiment in the present study is presented in Supplementary Material (Supplementary Figure 1).

#### Groundwater Containing Artificial Addition of Mineral Salts as the Inoculum

Ten grams of DW GAC loaded with sucrose, lactose, and LB medium were added into separate flasks containing 450 ml ground water and 50 ml 10× sterile mineral medium [1.5 g/L KH_2_PO_4_, 9.0 g/L Na_2_HPO_4_∙12H_2_O, 3 g/L (NH_4_)_2_SO_4_, 0.01 g/L CaCl_2_∙12H_2_O, 0.5 g/L MgSO_4_ (pH 6.8)] (Lee et al., 2006). Ten grams of DW new GAC was used as a control. Experiments were otherwise conducted as above (Supplementary Figure 1).

### Continuous-Flow Column Experiment

#### Reactor Lay-Out and Operation

The schematic presentation and photograph of the bench-scale GAC reactor system are shown in Supplementary Material (Supplementary Figures 2, 3). The system comprised 12 Teflon (polytetrafluoroethylene, PFTE) flow-through hollow cylinder reactors connected in parallel. Each reactor was filled with 0.124 L GAC (FILTRASORB 400) with a diameter of 0.55–0.75 mm and a density of 540 g/L to create a GAC filter. The DW of GAC in each filter was 66.96 g. GAC particles with substrate loading (sucrose, lactose, and LB medium) and without loading (control) were filled into triplicate reactors for each condition. The GAC filters were operated for 64 days in continuous up-flow mode with pressurized groundwater [described in section Water Quality of the Inoculum (Groundwater)] as the influent. Flow rate was controlled by valve adjustment and was set at 70 ml/min and a filtration velocity was 3.3 m/h. The empty bed contact time (EBCT) of each filter was 1.8 min.

#### Sample Collection

Water samples were collected from influent and filter effluents on operational Days 1, 2, 3, 4, 8, 16, 24, 32, 40, 46, 57, and 64 to analyze suspended bacterial concentrations. The influent samples on operational Days 4, 8, 16, 32, and 64 were collected for analysis of microbial communities. GAC samples (1 g wet weight each) were collected from the top of carbon layer of each filter on operational Days 4, 8, 16, 32, and 64 for quantification and characterization of biofilm biomass and microbial communities.

### Suspended Bacterial Concentrations Measurements With Flow Cytometry

The total cell concentration (TCC) in the water samples was measured with SYBR Green I staining and flow cytometry (FCM) as described previously in detail (Hammes et al., 2008; Prest et al., 2013). The intact cell concentration (ICC) in the water samples was measured with SGPI staining and FCM as described previously in detail (Nescerecka et al., 2018).

### Quantification of Biomass on GAC Particles

Granular activated carbon samples were treated as described as follow: 1 g wet GAC sample and 10 ml nanopure water were added into a 15 ml sterile tube. The entire biofilm suspension was sonicated with a BandelinSonorex device (Sonopuls HD 2200, BandelinSonorex, Rangendingen, Germany) with a needle at 50% intensity with 50% power for 30 s. The recovery (%) of biomass from solid sample by FCM for one cycle of high energy sonication (HES) accounted for about 41% (Vignola et al., 2018). TCC and ICC of bacteria suspension were monitored by FCM as described in section Suspended Bacterial Concentrations Measurements With Flow Cytometry.

### Microbial Diversity Analysis

#### DNA Extraction

DNA was extracted from biofilm and water samples. For the biofilm samples, the remaining volume of biofilm suspension obtained from GAC samples as described in section Quantification of Biomass on GAC Particles was filtered using sterile techniques on a 0.22 μm polycarbonate Nucleopore® membrane filter (47 mm diameter, Whatman, Kent, United Kingdom). For the influent water samples, 1 L of groundwater (i.e., the inoculum) was directly filtered using the same method. The filters were stored in 5 ml tubes at −20°C before DNA extraction with the Power Water DNA Isolation Kit (MoBio Laboratories, Inc., Carlsbad, Canada) according to manufacturers’ instructions.

#### PCR Amplification

Approximately 1 ng of DNA extract from each sample was subjected to PCR amplification. The V3–V5 region of the bacteria 16S rRNA gene was PCR amplified (95°C for 5 min, followed by 29 cycles at 98°C for 20 s, 51°C for 15 s, and 72°C for 15 s, and a final extension at 72°C for 5 min) using modified universal bacterial primers Bakt_341F and Bakt_805R, which were adapted with a tail to facilitate binding of Nextera adapters (Supplementary Table 1). Index PCR was performed (95°C for 3 min, followed by 10 cycles at 95°C for 30 s, 55°C for 30 s, and 72°C for 30 s and a final extension at 72°C for 5 min) to add the Nextera XT v2 Index Kit adapters (Illumina) to the amplicon (Supplementary Table 2). Amplicon PCR and Index PCR reactions were both performed in duplicate in a 25 μl reaction mixture containing Kapa HiFi HotStart ReadyMix (Kapa BioSystems) primers and template DNA. After each PCR reaction, products were purified using the Agencort® AMPure XP system (Beckman Coulter, Inc., Bera, Canada). Each product was quantified using the Qubit System (Thermo Fisher Scientific). Using these concentrations, all the samples were diluted to the same concentration (4 nM) using 10 mM Tris (pH 8.0) and then pooled together.

#### Illumina MiSeq Sequencing

The pooled sample was normalized before subjecting it to running on the MiSeq platform using a MiSeq Reagent Kit v2 (300-cycles, #MS-102-2002) according to manufacturer’s protocol. All sequencing was done at the Genetic Diversity Centre (GDC) of ETH, Zurich. The raw reads were deposited into the NCBI Sequence Read Archive (SRA) database with the Accession Number: SRP286279, BioProject ID: PRJNA667186.

#### Processing of Sequencing Data

Sequences were merged, trimmed, filtered, and clustered into operational taxonomical units (OTUs) according to several algorithms. Quality control was performed with Fast QC v.0.7. Reads were merged with FLASH v1.2.9, with minimum overlap of 15, maximum overlap of 200, and max mismatch density of 0.3 (Magoč and Salzberg, 2011). Sequences were trimmed with cutadapt v1.4, with an error rate of 0 (Martin, 2011). Quality filtering was performed with PRINSEQ-lite v0.20.4, with a size range of 450–550 bp, a minimum mean quality score of 25, no ambiguous nucleotides, and a GC range of 20–80 (Schmieder and Edwards, 2011). OTU clustering was performed with usearch v9.0.2132, with an identity cutoff of 97%, abundance sorting of 2, and chimera filtering was performed (Edgar, 2010). Sequences were identified according to the Greengenes v.13.5 (DeSantis et al., 2006). In R, phyloseq (McMurdie and Holmes, 2013) was used for processing. Libraries were rarefied to 65,025 sequences per sample, resulting in a total of 13,659 OTUs. The adonis function from the vegan package (Oksanen et al., 2013) was used to relate experimental conditions to microbiome composition.

### Absolute Abundance Calculation

The absolute abundance was calculated by multiplying the relatively abundance from high-throughput sequencing (above) with the total cell concentration (cells per gram of the corresponding GAC sample) from flow cytometric data as described in previous studies (Props et al., 2017; Vandeputte et al., 2017; Barlow et al., 2020; Jian et al., 2020).

### Water Quality Analysis

The influent water samples were measured for NH_4_^+^-N, total nitrogen (TN), total phosphate (TP), and total organic carbon (TOC). NH_4_^+^-N was detected by spectrophotometry after Berthelot’s reaction. TP was also determined using spectrophotometric method. The TOC and TN were detected using a TOC analyzer equipped with a TN measuring unit (TOC-VCPH, Shimadzu, Japan). DOC was determined by the TOC analyzer after filtration using a 0.45 μm cellulose acetate membrane.

## Results

### Substrate Selection

Acetate, glucose, sucrose, lactose, and LB medium were initially selected as the primary carbon substrates. The five substrates had different adsorptive characteristics with GAC (Supplementary Figure 4). The order of maximum adsorption (mg-C/g DW GAC) for the different substrates was acetate (1.09 ± 0.19) < glucose (2.83 ± 0.01) < LB medium (4.29 ± 0.06) < lactose (5.42 ± 0.11) < sucrose (5.68 ± 0.00). Desorption characteristics also differed considerably. Only an extremely small fraction of LB medium, lactose, and sucrose desorbed after 24 h (less than 0.2 mg-C/g DW GAC). The desorbed fraction of lactose from GAC was the lowest (0.07 ± 0.01 mg-C/g DW GAC). In contrast, a larger fraction of acetate (0.23 ± 0.01 mg-C/g DW GAC) and glucose (1.37 ± 0.01 mg-C/g DW GAC) desorbed from GAC when exposed to clean water. The result indicated that acetate and glucose were weakly adsorbed onto GAC. The order of the total desorbed amount (i.e., difference in amount between adsorption and desorption; mg-C/g DW GAC) for the five substrates was sodium acetate (0.86) < glucose (1.45) < LB medium (4.16) < lactose (5.35) < sucrose (5.55). Therefore, sucrose, lactose, and LB medium were selected as carbon substrates in the subsequent experiments in this study.

### Batch Experiments

#### Groundwater as the Inoculum and Water Matrix

**Figure 1:**
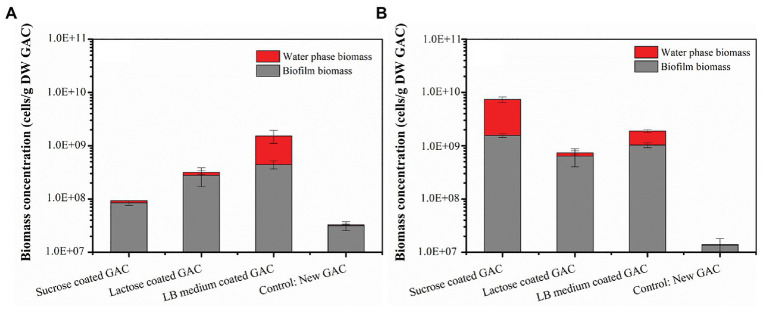
Total biomass in the different substrate-loaded GAC experiments without mineral salts **(A)** and with mineral salts **(B)** on Day 5. Water phase biomass (cells/ml) is expressed relative to the GAC (cells/g DW GAC) to allow comparison on the same graph as the biofilm data. Water phase biomass (cells/g DW GAC) was calculated through multiplying total cell concentration of the water sample (FCM data) by the volume of water in a flask and then dividing by the mass (dry weight) of activated carbon in the flask. Error bars indicate SD on triplicate experiments.

When the loaded GAC was incubated with groundwater without additional nutrients, the resulting growth did not necessarily match the growth potential of the adsorbed carbon. Figure 1A illustrates the biomass in the different loaded GAC experiments, including both water phase and biofilm biomass, cultivated in groundwater for 5 days. The order of total biomass (cells/g DW GAC) in all experiments was control [(3.28 ± 0.58) × 10^7^] < sucrose loaded GAC [(9.29 ± 0.82) × 10^7^] < lactose loaded GAC [(3.16 ± 1.02) × 10^8^] < LB medium loaded GAC [(1.53 ± 0.34) × 10^9^]. The result shows that substrate-loaded GAC enriched bacterial growth between 3–46 times compared to the growth in the control (unloaded GAC). The results indicated that substrate-loaded GAC enhanced initial surface colonization in a short incubation period (5 days).

#### Groundwater With Artificial Addition of Mineral Salts

When inorganic nutrients were supplemented in the water matrix, more growth was observed in the substrate-loaded GAC experiments. The total biomass in the experiments cultivated in groundwater containing artificial addition of mineral salts for 5 days are shown in Figure 1B. With the addition of mineral salts, the inorganic nutrients (N, P) were no longer limiting. The growth of bacteria in all experiments reached stationary phase on Day 5 (Supplementary Figure 6). Under these conditions, the order of total biomass (cells/g DW GAC) in the four experiments was control [(1.40 ± 0.45) × 10^7^] < lactose loaded GAC [(7.39 ± 1.84) × 10^8^] < LB medium loaded GAC [(1.89 ± 0.19) × 10^9^] < sucrose loaded GAC [(7.39 ± 0.79) × 10^9^]. The total biomass in the sucrose-loaded GAC experiment was 80 times higher than under the nutrient limited conditions (i.e., no mineral salts added). The total biomass in the lactose-loaded GAC experiment was two times higher than in the nutrient limited condition. However, the values for LB medium loaded GAC experiments with and without nutrient limitation were almost the same. All data combined, the pre-loading of GAC with organic carbon substrates resulted in biofilm growth of at least 23–225 times compared to unloaded GAC, indicating that this approach can be used to rapidly colonize the GAC particles with bacteria under defined incubation conditions.

#### The Microbial Community on GAC Particles in the Batch Experiments

**Figure 2:**
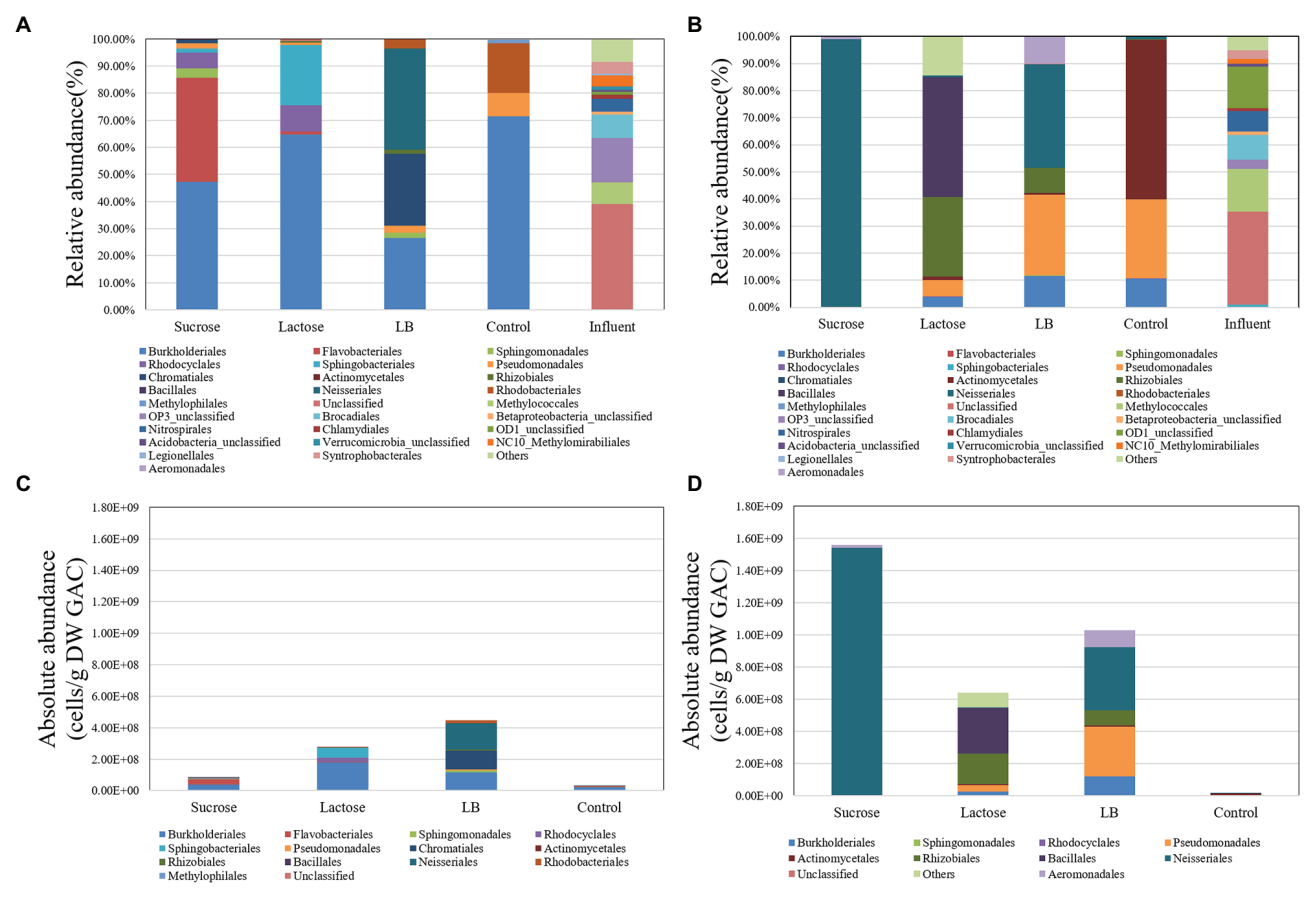
The microbial communities (relative and absolute abundance) on the level of “order” in the GAC particles and the initial inoculum in the different substrate-loaded GAC batch experiments without mineral salts [nutrient limited; **(A,C)**] and with mineral salts [nutrient sufficient**; (B,D)**] on Day 5.

The microbial communities in the initial inocula of the two batch experiments were similar but differed considerably from the communities on the GAC after 5 days incubation (Figures 2A,B), showing that the selection occurred. In both experiments, different organic carbon substrates selected for clearly different microbial communities in the stationary phase. Moreover, the inorganic nutrients in the two batch experiments were substantially different, and the resultant microbial communities on Day 5 also had substantial differences from each other (Figure 2). Under the inorganic nutrient-limited conditions (Figures 2A,C), Proteobaceria (specifically Burkholderiales) dominated across all carbon conditions, albeit at different relative abundances. However, under excess inorganic nutrient conditions (Figures 2B,D), the communities diverged more, based on the type of carbon added. Here, sucrose loaded GAC was completely dominated by OTUs from the order Neisseriales, while lactose and LB loaded GAC showed Rhizobiales, Bacillales, and Pseudomonales as additional dominant orders. All of these orders have genera isolated previously from freshwater environments, and therefore, their enrichment on common substrates was not surprising. From Figure 1, it is evident that some attachment and growth occurred in the control samples as well. The data in Figure 2 shows that this also resulted in considerable community changes relative to the inoculum samples. A community shift with addition of inorganic nutrients to the control experiments occurred even though the total growth did not increase, suggesting that the change in inorganic nutrients influenced the overall ecology. From the data in Figures 2A–D we conclude that batch growth of a complex groundwater inoculum on GAC pre-loaded with different substrates resulted in substantial biomass increase relative to an untreated control and selection of different microbial communities across all treatments.

### Continuous Experiment

#### Dynamics of Water Phase Biomass Concentration

**Figure 3:**
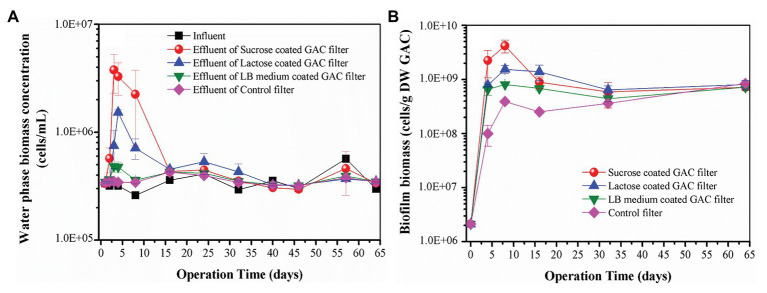
**(A)** Water phase biomass concentration in influent and effluent of different substrate-loaded GAC filters; **(B)** biofilm biomass of different substrate-loaded GAC filters. Error bars indicate SD on triplicate experiments.

In the flow-through GAC filters, water phase biomass concentration dynamics were monitored over time. Within 16 days of operation, a very clear difference in the water phase biomass concentrations in the effluent occurred across all filters. Figure 3A illustrates the water phase biomass concentrations of the influent and effluent of four treatments during 64 days of operation. Groundwater flowed continuously into the filters, providing a continuous inoculum providing both bacteria and inorganic nutrients to the GAC particles. The water phase biomass concentrations in influent were stable at (3.47 ± 0.79) × 10^5^ cells/ml, 60 ± 13% of which were intact bacteria (Supplementary Figure 7). However, differences in water phase biomass concentrations in the effluent of all the GAC reactors were apparent (Figure 3A). The water phase biomass concentration in the effluent of the control filter (i.e., stable at (3.55 ± 0.31) × 10^5^ cells/ml) was consistent with that of the influent. However, during the beginning of operation, the effluent biomass concentration peaked for each of the substrate-loaded GAC filters, with different magnitudes. From Day 1 to Day 16, the effluent biomass concentration of sucrose- and lactose-loaded GAC filters peaked at (3.78 ± 1.50) × 10^6^ and (1.53 ± 0.09) × 10^6^ cells/ml, respectively. During the operation of Day 1 to Day 8, the effluent biomass concentration of LB-medium-loaded GAC filters peaked at (4.83 ± 0.06) × 10^5^ cells/ml. After 16 days of operation, the effluent biomass concentrations of all the GAC filters stabilized (at approximately 3.66 × 10^5^ cells/ml), following a similar pattern to the influent biomass concentration.

In addition, the average difference in TOC between the influent and effluent of the three kinds of substrate loaded GAC filters ranged 0.06–0.17 mg/L (only accounting for 5–12% of the average influent TOC concentration), while the average value of the control filter was 0.17 mg/L. This result suggested that the addition of carbon before reactor initialization had no significant influence on effluent TOC concentrations and that little, if any, of the groundwater TOC was bioavailable (Supplementary Figure 8).

#### Dynamics of Biofilm Biomass

During the operation of 64 days, the dynamic trends of biofilm biomass in the substrate-loaded GAC filters were similar. Figure 3B illustrates the dynamics of biofilm biomass from all the GAC filters. The biofilm biomass increased dramatically from Day 0 to Day 8, subsequently decreased during Day 8 to Day 32, finally increased slightly to approximately the same value [(7.8 ± 0.62) × 10^8^ cells/g DW GAC]. The biofilm biomass for sucrose, lactose, and LB-medium-loaded GAC filters peaked at (4.19 ± 1.10) × 10^9^, (1.55 ± 0.26) × 10^9^, and (8.11 ± 0.36) × 10^8^ cells/g DW GAC on Day 8, which were 11, 4, and 2 times higher than that of the control filter, respectively. The results demonstrate that substrate-loaded GAC resulted in faster colonization and growth on the GAC, thus potentially shortening the start-up times of the GAC filters.

#### Microbial Community Dynamics

**Figure 4:**
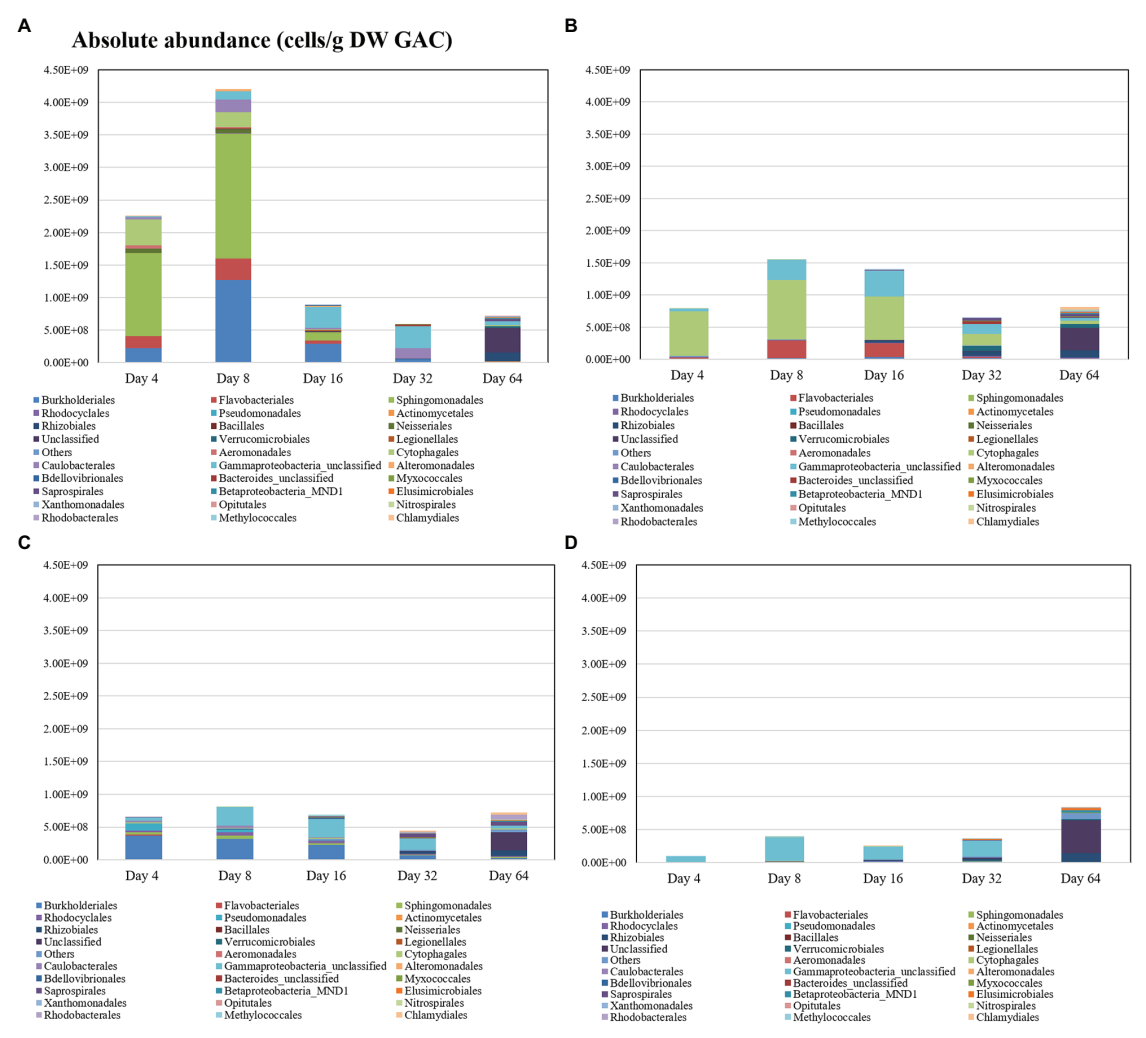
Absolute abundance of dominant orders in the continuous-flow GAC biofilters with **(A)** sucrose, **(B)** lactose, **(C)** LB medium, and **(D)** non-loaded (control) additions.

In the flow-through GAC filters, microbial community dynamics were monitored over time, with biofilm samples taken at Days 4, 8, 16, 32, and 64. In this period, a very clear shift in the microbial community occurs across all filters (Table 2 and Figure 4). Table 2 shows the dynamics of alpha diversity indices of substrate-loaded GAC filters during operation time. At the beginning of the operation time, the alpha diversity was the highest in the control filter (Shannon index: 3.72 ± 0.84). However, when a carbon source was added before reactor initialization, richness started very low (i.e., less than 2.8 of Shannon index). The diversity of substrate-loaded GAC filters increased gradually over time. At the end of the experiment, biofilms of most reactors had very similar alpha diversity. In each set of GAC reactors, the starting community was distinct on the order level, but this faded over time as richness increased (Figure 4 and Supplementary Figure 9). When GAC was pre-loaded with sucrose, Sphingomonadales (57%), Cytophagales (17%), Burkholderiales (10%), and Flavobacteriales (8%) dominated early on, but they were all below 2.5% relative abundance by Day 64. When GAC was pre-loaded with lactose, Cytophagales (88%) dominated at the beginning and then gradually decreased to less than 10% by Day 64. When GAC was pre-loaded with LB medium, Burkholderiales (56%) and Pseudomonadales (16%) dominated at the start but both decreased to below 3% by Day 64. The control without any carbon addition to the GAC was dominated by unclassified Gammaproteobacteria (76%) until Day 32. On Day 64, all treatments were dominated by unclassified OTUs, which is similar to the original groundwater (Figure 2).

**Table 2:** Diversity indices of substrate-loaded GAC filters and the inoculum.

	Operation time (day)	Observed OTUs	Chao1	Shannon	Simpson
Inoculum (groundwater)	4, 8, 16, 32, 64	6,349 ± 290	7,428.32 ± 435.14	7.27 ± 0.04	1.00 ± 0.00
Sucrose loaded GAC filter	4	414 ± 246	566.37 ± 318.78	1.80 ± 0.55	0.66 ± 0.15
	8	332 ± 87	470.58 ± 137.52	1.90 ± 0.24	0.71 ± 0.06
	16	664 ± 162	821.98 ± 242.81	2.93 ± 0.09	0.86 ± 0.02
	32	1,164 ± 78	1,403.51 ± 65.41	3.81 ± 0.08	0.92 ± 0.01
	64	1,756 ± 109	2,110.71 ± 179.62	4.14 ± 0.22	0.90 ± 0.03
Lactose loaded GAC filter	4	995 ± 85	1,275.56 ± 128.83	1.15 ± 0.07	0.32 ± 0.01
	8	628 ± 93	909.41 ± 130.78	1.78 ± 0.42	0.68 ± 0.13
	16	657 ± 144	871.46 ± 219.30	2.08 ± 0.06	0.74 ± 0.02
	32	826 ± 137	1,026.02 ± 193.69	3.16 ± 0.35	0.88 ± 0.05
	64	1,756 ± 269	2,183.89 ± 408.07	4.10 ± 0.34	0.92 ± 0.03
LB loaded GAC filter	4	814 ± 49	1,109.48 ± 45.79	2.79 ± 0.12	0.87 ± 0.01
	8	783 ± 79	1,043.72 ± 131.45	2.66 ± 0.10	0.83 ± 0.02
	16	1,110 ± 396	1,457.85 ± 597.85	3.19 ± 0.27	0.86 ± 0.02
	32	1,324 ± 62	1,647.82 ± 65.00	4.08 ± 0.05	0.94 ± 0.01
	64	1,519 ± 145	1,902.63 ± 226.14	3.99 ± 0.23	0.91 ± 0.02
Control filter	4	3,581 ± 637	4,520.00 ± 818.92	3.72 ± 0.84	0.74 ± 0.13
	8	2,288 ± 77	2,965.84 ± 127.34	2.14 ± 0.08	0.44 ± 0.02
	16	2,452 ± 144	3,092.27 ± 183.51	3.07 ± 0.03	0.66 ± 0.01
	32	2,021 ± 56	2,475.07 ± 91.39	3.70 ± 0.10	0.87 ± 0.01
	64	1,881 ± 21	2,322.39 ± 105.87	3.63 ± 0.05	0.87 ± 0.01

The communities at Day 64 were also remarkably similar across all four kinds of GAC filters, which is consistent with the biofilm biomass measured on GAC particles. The difference in communities can also be visualized with non-metric multi-dimensional scaling (NMDS; Supplementary Figure 10). Samples from the different types of reactors became more similar with time. Considering only the biofilms in these flow-through reactors, the kind of loaded substrates accounted for 30% of community variation, time accounted for 41%, and the interactions between the two factors (time and loaded substrates) accounted for 20% of community variation (adonis, *p* < 0.001).

## Discussion

### Initial Colonization

#### Carbon Source Selection and Nutrient Limitations

We pre-loaded new GAC with different carbon sources and followed initial microbial community colonization and selection, as well as the long-term stability of the selected communities. Substrate loading is known to enhance biological activity in biofilters (Li, 2016; Qin et al., 2017; Zhao et al., 2019). However, to our knowledge, the approach to pre-load new GAC with substrates in order to facilitate initial selection and growth of biofilm communities remains largely untested. For this proof-of-concept study, we specifically selected relatively common substrates (sucrose, lactose, and LB medium). The purpose was not to select for any specific degradation pathway *per se* but to demonstrate that substrate pre-loading of GAC leads to rapid biofilm formation and differences in community composition.

In all batch experiments, the total biomass obtained was considerably lower than expected for the amount of carbon adsorbed to the GAC (Figure 1A). The total amount of adsorbed carbon on GAC loaded with sucrose, lactose, and LB medium was 54.0 ± 1.5, 54.7 ± 0.4, and 46.4 ± 2.3 mg-C, respectively. Based on a conversion factor of 10^7^ bacteria grown per μg of assimilable organic carbon (AOC; Hammes and Egli, 2005), the expected total cell counts were between 4.6 × 10^11^ and 5.5 × 10^11^ cells. The theoretical values were therefore one or two orders of magnitude higher than the observed values. The difference between observed and theoretical values might be due to (i) limitation of nitrogen and phosphorus, (ii) incomplete biodegradation of carbon adsorbed on GAC, (iii) limited extraction efficiency of bacteria from GAC samples by the sonication method (HES), and/or (iv) a difference in yield from that assumed with general conversion factor (for detailed discussion, see Supplementary Text 1).

When inorganic nutrients were supplemented in the water matrix in the batch experiment, the total biomass in sucrose pre-loaded GAC experiments increased by 78.5 times, whereas it increased by only 1.3 times in lactose pre-loaded GAC experiments. Rodrigues and colleagues found that the yields of biomass growth per substrate consumption of sucrose by *Lactococcus lactis* 53 and *Streptococcus thermophilus* A, both having lactase enzyme, were 1.6 and 2.5 times, respectively, compared to the values of lactose under nutrient sufficient conditions (Rodrigues et al., 2006). It is possible that bacteria would require more energy for expressing lactase. Hence, the large increase for sucrose loaded GAC when the mineral salts were added compared to the only slight increase for lactose loaded GAC, which could be attributed to more energy cost of expressing lactase. The total growth did not increase in the control experiment, even though the C/N/P ratio indicated a nitrogen limitation. This likely indicates that the carbon measured here was not bioavailable. In the system, carbon was the primary nutrient limitation, which could be overcome with carbon substrate addition, while nitrogen and phosphorous were the secondary nutrient limitations, which could be overcome with mineral salt addition.

Furthermore, the actual biofilm biomass in the sucrose pre-loaded GAC filters on Day 8 in the continuous experiment [(4.20 ± 1.11) × 10^9^ cells/g GAC] was the closest to the theoretical value (9.9 × 10^10^ cells/g GAC). This result was consistent with the result in the nutrient-rich batch experiment (i.e., groundwater containing artificial addition of mineral salts as the inoculum and water matrix), suggesting that the nutrient level in the flow-through GAC filters was adequate. Carbon was supplied in excess on the GAC, while nitrogen, phosphorous, and other nutrients were supplied continuously in low amounts from the groundwater (C:N:P ratio was 100:274:2, with sufficient N and P elements) and could have accumulated in biofilm. Even though the amount of sucrose, lactose, and LB medium adsorbed by GAC in the flow-through GAC filters were 9.93, 9.96, and 9.19 mg-C/g DW GAC, approximately two times compared to the batch experiments, the biofilm biomasses in the flow-through substrate-loaded GAC filters were all considerably less than the values in batch experiments. This result could be due to the fact that detached and/or dead bacteria from GAC particles are continuously removed by flowing effluent.

#### Initial Biofilm Formation

Nutrients likely influenced colonization through selection. The data in the above sections showed that biomass on GAC increased significantly under conditions with sufficient nutrients. In this study, either in batch or continuous experiment, the biofilm on substrate-loaded GAC reached 10^7^–10^9^ cells/g DW GAC only after 5–8 days, which equaled to 10^7^–10^8^ cells/cm^3^ [(calculated based on cells per g DW GAC and the loading density (*ρ* = 0.425 g/cm^3^) of GAC], which is much quicker than that observed in the control systems without substrate pre-loading (at least 33 days). However, these values are still one to two orders of magnitude lower than previously measured cell concentrations by FCM in drinking water biofilters (10^9^–10^10^ cells/cm^3^; Velten et al., 2007; Lautenschlager et al., 2014; Vignola et al., 2018). Thus, in the sucrose, lactose, and LB-medium-loaded GAC experiments, the bacteria, which possess metabolic traits of the specific carbon source, rapidly fill the empty niches (surface of GAC particles). The initial microbial communities were established successfully through resource utilization, which was defined as selection (Kinnunen et al., 2016). The microbial communities on sucrose, lactose, LB-medium-loaded GAC and control GAC were significantly different. Furthermore, the differences of initial richness between substrate-loaded GAC filters and control filter in the continuous experiment were significant, demonstrating that only few species could specialize in consuming that carbon source or that few of the fastest competitors were able to dominate the niche space and exclude others. This result was consistent with the batch experiments, indicating that selection played a significant role in initial microbial community establishment.

A bacterium transfers from the inocula (water phase) onto the surface of GAC particles, which is a dispersal process (Kinnunen et al., 2016). Dispersal is likely a determinant of colonization. In the batch experiments, with 150 rpm shaking speed, bacteria will have ample opportunities to immigrate into the community on GAC particles (i.e., attachment) or emigrate out (i.e., biofilm detachment). In comparison with batch experiments, the initial microbial communities (Day 4) in the flow-through filters also had substantial differences, even though the influent microbial communities were the same (Figure 2 and Supplementary Figure 11). The nutrition supply method and the inoculation method in batch experiments were substantially different from that of the flow-through filters. Inoculum (bacteria) and nutrients in water matrix (groundwater) were added all at once into the batch experiments but were continuously supplied for the flow-through filters. Batch experiments were closed systems, so the dead or/and detached bacteria from GAC particles remained in the system. For the flow-through filters, dead, or/and detached bacteria could move out of the filter with water flow. The results demonstrated that different conditions of dispersal (continuous or limited) and selection (nutrient supply) processes led to different initial colonization as previously observed (Besemer et al., 2007).

### Microbial Community Succession in GAC Filter

For all flow-through filters, the biofilm communities were remarkably different to that of the influent groundwater microbial community (Figure 2; Supplementary Figure 11). They also had lower alpha diversities than the influent water (Shannon index: 7.27 ± 0.04 in the water throughout the experiment), suggesting that biofilm formation on GAC particles, in general, was a highly selective environment. From the data in Figure 4, it is evident that each treatment selected for different bacterial communities after 4 days of operation, presumably a result of selective attachment and growth (Figure 3). During the subsequent 64 days of operation, the microbial communities in all treatments increased in diversity and became more similar to each other and to the influent water. This is ascribed to the lack of additional growth supporting nutrients in the influent, continuous dispersal, stochastic drift, and attachment/detachment of bacteria on the GAC.

**Figure 5:**
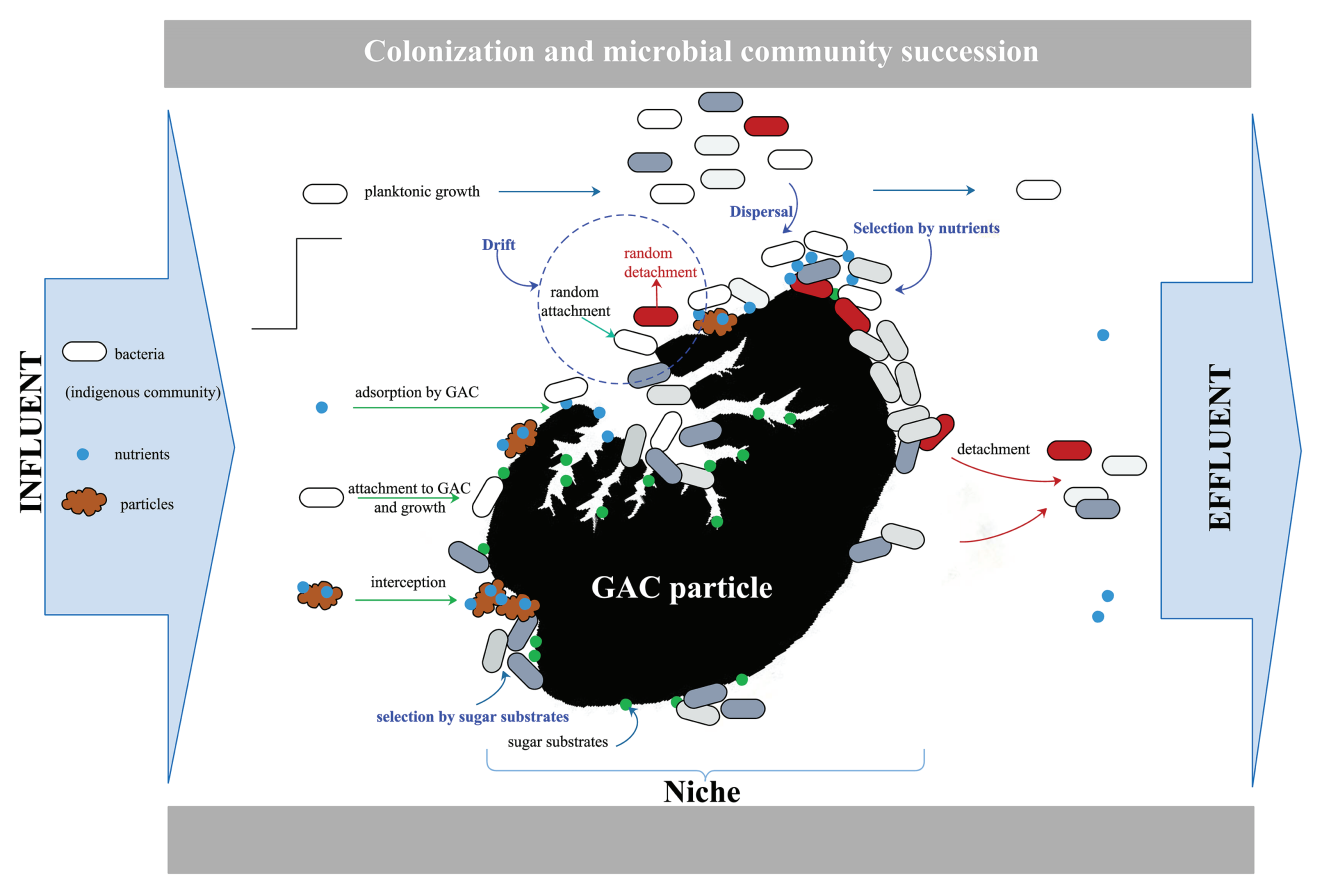
Conceptual overview of microbial dynamics on a GAC particle in a flow-through GAC filter.

Figure 5 illustrates the conceptual overview of microbial dynamics on the GAC particle in a flow-through GAC filter. The constant influent, which contains bacteria (groundwater community), nutrients (carbon, nitrogen, and phosphorus source), and particles, flows through GAC particles with a specific sugar substrate (empty niches and sufficient nutrients). Bacteria disperse with the flow and attach randomly onto the surface or pores of the GAC particle. A resident microbial community on the GAC particle establishes rapidly, selected for by growth on the specific sugar substrate (data on Day 4 in Figure 4), resulting in an increase in biomass (data on Day 8 in Figure 4). Altogether, this enriches the initial colonization on GAC filters.

However, the resident community cannot maintain stability over time when challenged by the experimental conditions given. The nutrients and bacteria in the influent constantly enter the system as the specific sugar substrate is consumed gradually by the resident community. The enriched biofilm biomass on the GAC particles decreases, and the dominant bacteria in the resident community gradually lose their ability to compete. The reasons for this phenomenon are diverse. Influent community is added to the resident communities *via* dispersal, and the relative abundances of the communities are then shaped by selection (consumption of added carbon substrate) and ongoing dispersal (with continuous introduction of a challenging community), as well as drift (stochastic effects), to drive community dynamics.

## Practical Implications

The findings of this study demonstrated that pre-loading organic carbon substrates on GAC could promote rapid initial colonization of biofilms with high coverage and that based on the substrates that are loaded, specific communities can be selected. However, the initial colonization selected by pre-loading sugar did not remain stable for prolonged operational periods due to continuous competition from the influent microbial community and the absence of additional growth supporting nutrients in the groundwater. While we focused in this study on colonization and selection using common carbon sources, future work should ideally investigate more specific/relevant substrates. BAC/GAC filtration is commonly used for removal of micro-pollutants from drinking water and wastewater (Kalkan et al., 2011; Chu et al., 2012). Targeted enrichment during initial GAC colonization could promote targeted pollutants removal (Gao et al., 2010). Thus, the manipulation of GAC biofilter biofilms by surface pre-loading shows some potential for future application. In this study, several sugars and LB medium were selected as the proof-of-concept carbon sources for the initial colonization and substrate loaded on GAC particles. However, these were neither major carbon sources (or pollutants) in the groundwater used as influent nor are they particularly interesting from a biodegradation perspective. The former may be one reason why the initial colonization was not maintained. In future studies, we will focus on specific micro-pollutants from drinking water and wastewater as the pre-loading carbon sources and focus on the long-term stability of the initial colonization.

## Data Availability Statement

The datasets presented in this study can be found in online repositories. The names of the repository/repositories and accession number(s) can be found at: NCBI SRA database with the Accession Number: SRP286279; BioProject ID: PRJNA667186.

## Author Contributions

WQ planned and conducted the experiments and wrote the manuscript. FH helped in planning the experiments, co-writing, and revising the article. All authors contributed to the article and approved the submitted version.

### Conflict of Interest

The authors declare that the research was conducted in the absence of any commercial or financial relationships that could be construed as a potential conflict of interest.
