# Supplementary material for: Substrate Pre-loading Influences Initial Colonization of GAC Biofilter Biofilms
Source: Front Microbiol. 2021 Jan 12;11:596156. doi: 10.3389/fmicb.2020.596156 (PMC7835318; doi:10.3389/fmicb.2020.596156)
Supplement: Supplementary file 1 [file Data_Sheet_1.pdf]

Supplementary Material

1 Supplementary Text, Figures and Tables

1.1 Supplementary Text

Supplementary Text 1. The explanation of the difference between observed and theoretical total biomass on GAC

(i) Firstly, the elemental molar C:N:P ratios in the experiments were calculated to assess whether the macronutrients were optimal for bacterial growth, using the data from the total amount of carbon adsorbed onto GAC, the DOC, NH_4^+-N and TP measurements. The optimal biochemical oxygen demand (BOD_5) to nitrogen to phosphorus ratio (BOD_5:N:P) for bacterial growth is approximately 100:5:1 (Baeza et al. 2016, Fan et al. 2004), equal to a carbon to nitrogen to phosphorus (C:N:P) mass ratio of 100:7:1, calculated by a converting factor of molar mass ratio of carbon element (12 g/mol) and oxygen element (16 g/mol). By setting carbon as 100, the C:N:P ratios in the sucrose and lactose loaded GAC experiments were both 100:4.8:0.03 based on TOC, TN, TP data (section 2.2) and pre-loaded amounts of carbon sources described above, indicating that N and P were the limiting factors for these conditions. However, LB-medium contains a multitude of sugars, amino acids, nucleic acids, etc., with 10 g tryptone per liter, 5 g yeast extract per liter, 10 g NaCl per liter (Ihssen and Egli 2005, Berney et al. 2006, Ihssen and Egli 2004). This indicates that LB-medium contains not only a carbon source, but also nitrogen and phosphorous sources. Thus, the C/N/P ratio in the LB-medium experiment was likely more optimal, contributing to the higher total bacteria numbers observed in that experiment (**Figure 1(a)**). It should be noted that the C:N:P ratio in the control experiment was 100:274:2, suggesting that N and P were not limited. However, the observed total cell count of the control experiment also only accounted 3.4 % for the theoretical value calculated by the amount of carbon source (9.6×10^9 cells). It is likely that the carbon present in the groundwater is not bioavailable. (ii) Secondly, for bacteria to grow, access to the adsorbed organic carbon is required. The amounts of adsorbed sucrose and lactose were higher than that for LB-medium (**Supplementary Figure 4(a)**), and the desorbed fraction of sucrose and lactose from GAC were extremely low (**Supplementary Figure 4(b)**). Furthermore, the water phase biomass in the sucrose and lactose loaded GAC experiments accounted for only 9 and 12 % of the total biomass in each experiment, respectively. However, the proportion of water phase biomass in the LB-medium loaded GAC experiment was as high as 71 %. These results indicated that the desorption of sucrose and lactose from GAC was more difficult than for LB-medium. Sucrose and lactose are small molecules in comparison with LB-medium. It is possible that smaller molecules adsorbed deep inside the activated carbon pore structure, making them less bioavailable (slow desorption, sorption hysteresis). After 5 days, not all adsorbed organic carbon was desorbed and/or consumed. Therefore, incomplete biodegradation of carbon adsorbed onto GAC likely

contributed to the observed differences between the observed total bacteria numbers and the expected theoretical values. (iii) Thirdly, a previous study reported that the extraction efficiency of bacteria from sand from water biofilters by 1 step HES (80s) was approximately 41 % (Vignola et al. 2018a). In this study, bacteria were extracted from GAC by high energy sonication for just 1 step (30s, details in section 2.6). The biomass for 1 step of HES accounted about 45 % the biomass for 10 steps of sonication (unpublished data). The data above showed that the extraction efficiency of bacteria from GAC samples by the sonication method was limited, which lead to lower observed total bacteria than theoretical predicted values. (iv) Fourthly, the conversion factor of 10^7 bacteria grown per μg of AOC is an empirical value. Previous studies have reported that yield values for different pure cultures growing on various carbon sources ranged between 2.9×10^6 and 1.2×10^7 CFU per μg of AOC (Van der Kooij 2003). Thus, there is at least a 4-fold variation in the conversion factor for different organisms and substrates. This may further explain why the observed amounts of bacteria in the experiments were 1-2 orders of magnitude lower than theoretical values.

Reference

Baeza, R., Jarpa, M., and Vidal, G. (2016). Polyhydroxyalkanoate Biosynthesis from Paper Mill Wastewater Treated by a Moving Bed Biofilm Reactor. Water Air Soil Poll. 227(9), 299.

Fan, Y., Wang, Y., Qian, P. and Gu, J. (2004). Optimization of phthalic acid batch biodegradation and the use of modified Richards model for modelling degradation. Int. Biodeter. Biodegr. 53, 57-63.

Ihssen, J. and Egli, T. (2005). Global physiological analysis of carbon- and energy- limited growing *Escherichia coli* confirms a high degree of catabolic flexibility and preparedness for mixed substrate utilization. Environ. Microbiol. 7, 1568-1581.

Ihssen, J. and Egli, T. (2004). Specific growth rate and not cell density controls the general stress response in *Escherichia coli*. Microbiology 150, 1637-1648.

Berney, M., Weilenmann, H., Ihssen, J., Bassin, C. and Egli, T. (2006). Specific growth rate determines the sensitivity of *Escherichia coli* to thermal, UVA, and solar disinfection. Appl. Environ. Microb. 72, 2586-2593.

Vignola, M., Werner, D., Hammes, F., King, L.C., Davenport, R.J. (2018a). Flow-cytometric quantification of microbial cells on sand from water biofilters. Water Res. 143, 66-76.

Van der Kooij, D. Assimilable Organic Carbon (AOC) in Treated Water: Determination and Significance. Hoboken: John Wiley & Sons Press (2003).

Supplementary Text 2. The description of the dynamics of intact bacteria in continuous experiment

Additionally, the dynamics of intact bacteria concentrations either in water phase or biofilm phase were basically similar to that of total bacteria concentrations (**Figure 3 and Supplementary Figure 7(a)(b)**). The proportions of intact bacteria accounting for the total bacteria on the GAC particles of sucrose, lactose, and LB-medium loaded GAC filters and control filter peaked at different operation time, i.e. on Day 4, Day 8, Day 8 and Day 16, respectively (**Supplementary Figure 7(c)**), suggesting that the growth periods of bacteria in different substrate loaded GAC biofilm were different. Growth period of bacteria was the shortest in sucrose loaded GAC filter, leading to the sharp decrease in biofilm biomass after Day 8.

1.2 Supplementary Figures

Supplementary Figure 1. Schematic overview of the two batch experiments: (a) Groundwater as inoculum and (b) Groundwater containing artificial addition of mineral salts as inoculum.

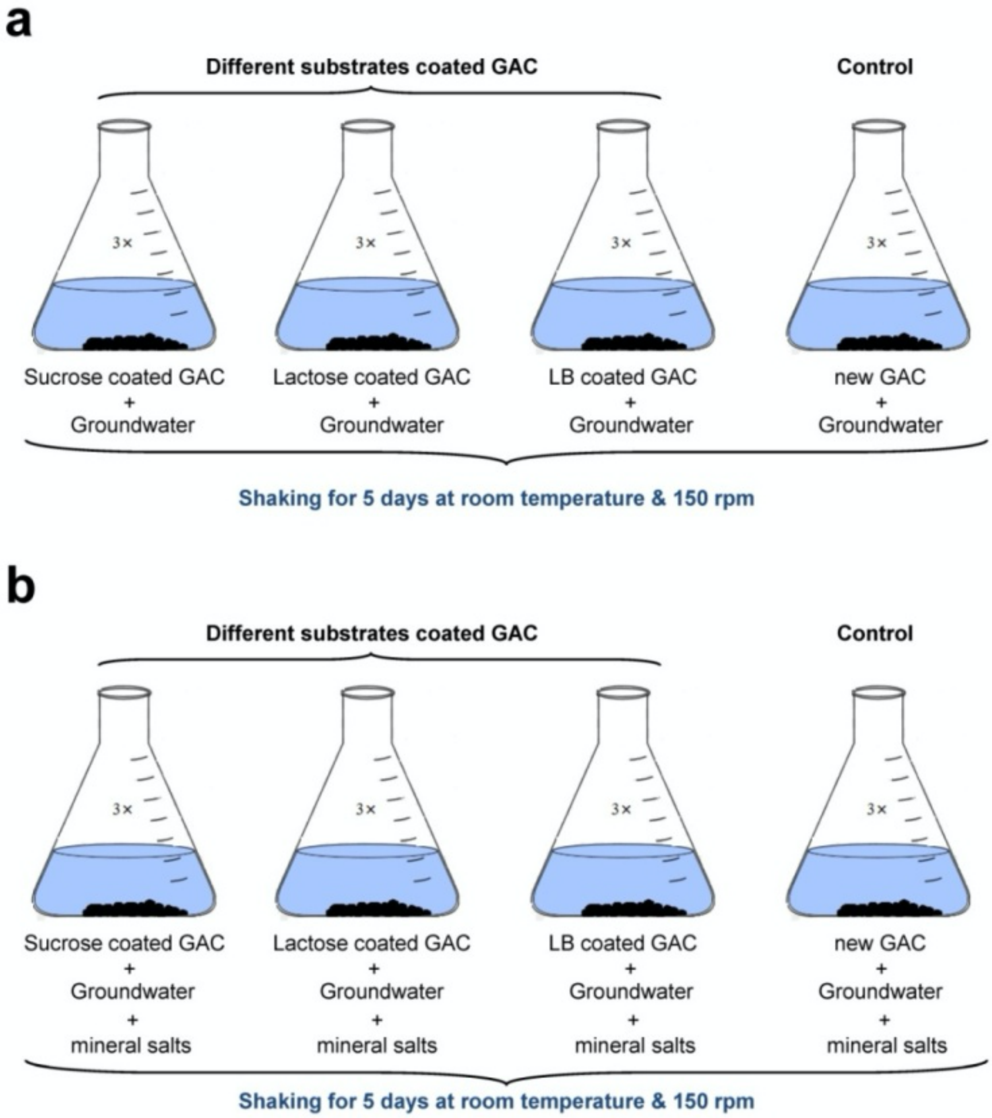

Supplementary Figure 2. Schematic presentation of the investigated bench-scale GAC filters.

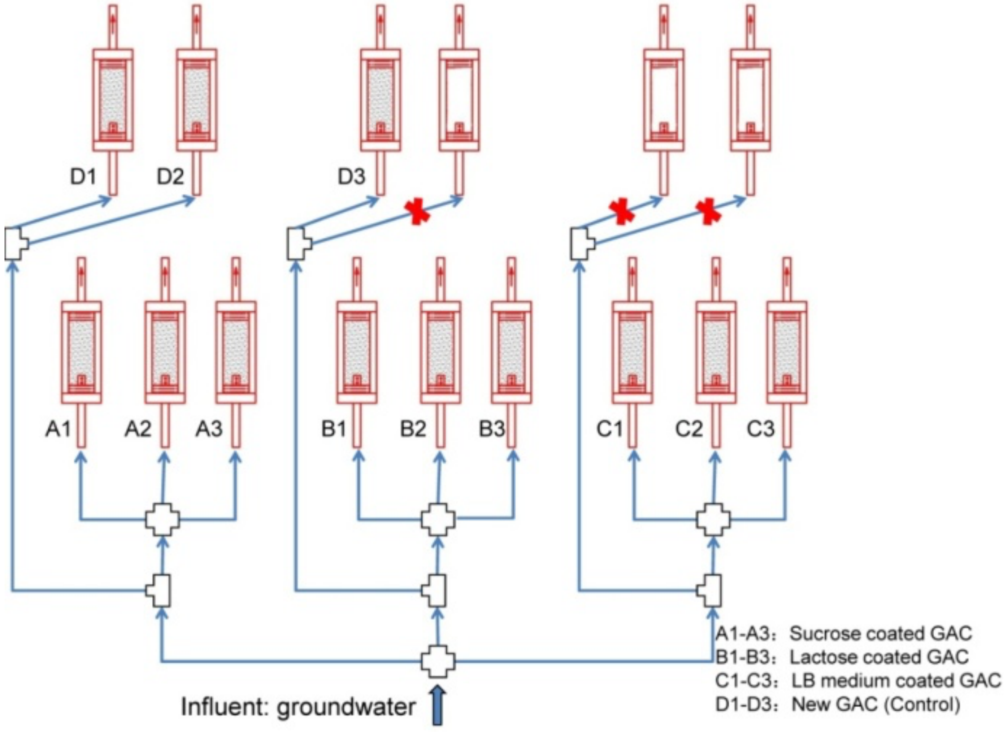

Supplementary Figure 3. Photo of the bench-scale GAC reactors.

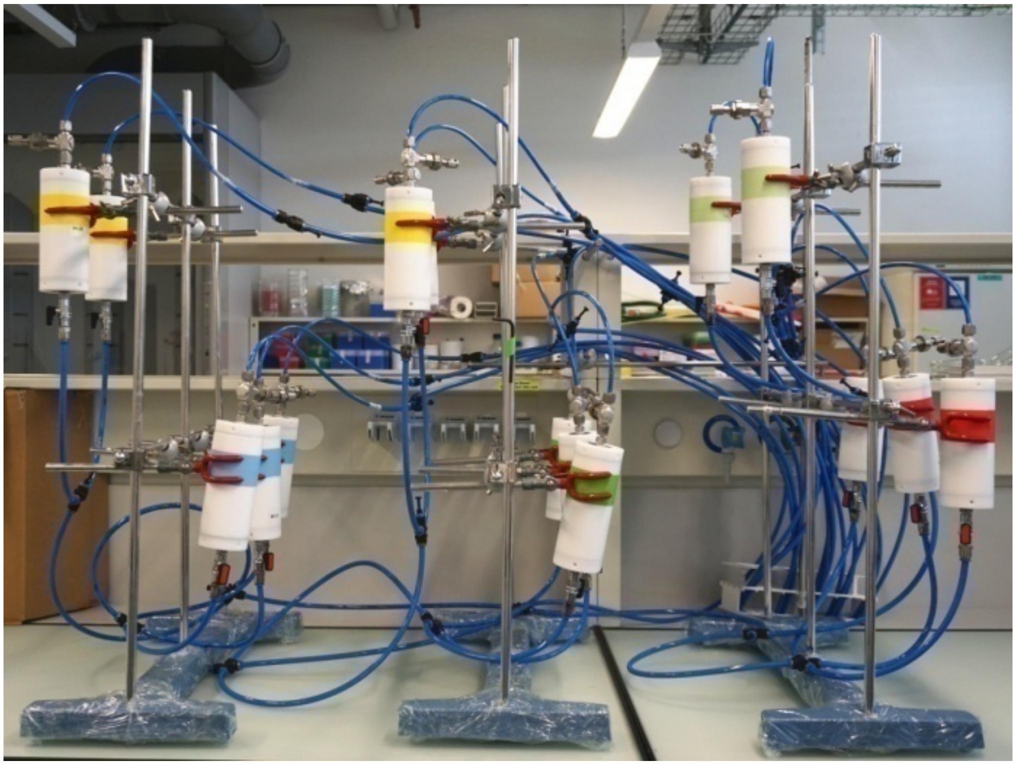

Supplementary Figure 4. (a) Adsorption characteristics of different substrates by GAC; (b) The desorbed fraction of different substrates from substrate coated GAC after 24h.

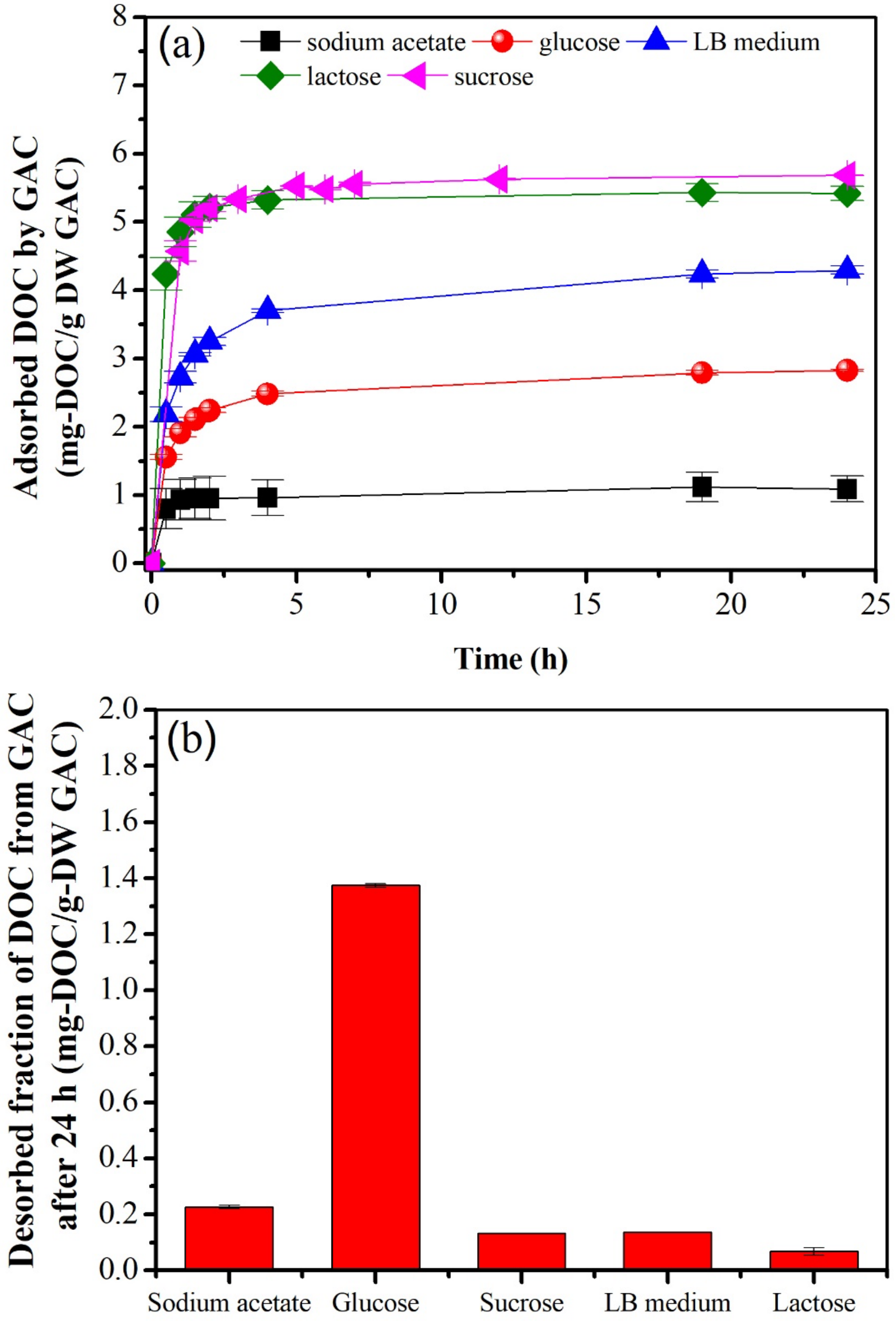

Supplementary Figure 5. Dynamics of suspended bacteria in the different substrate coated GAC system during 5 days.

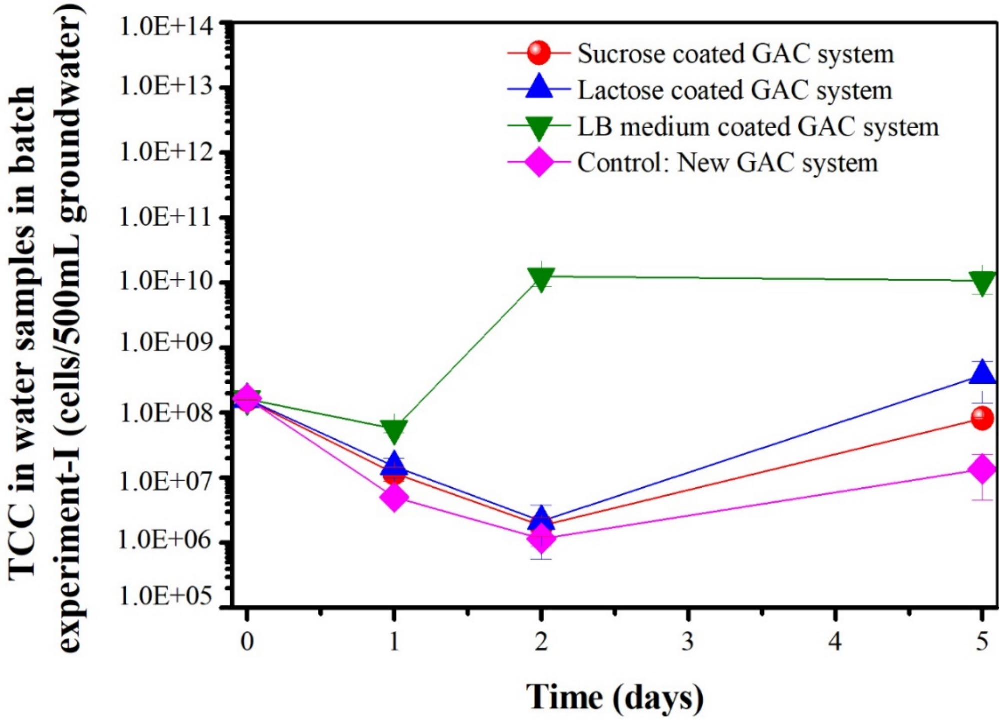

Supplementary Figure 6. Dynamics of suspended bacteria in the different substrate coated GAC system with mineral salts during 5 days.

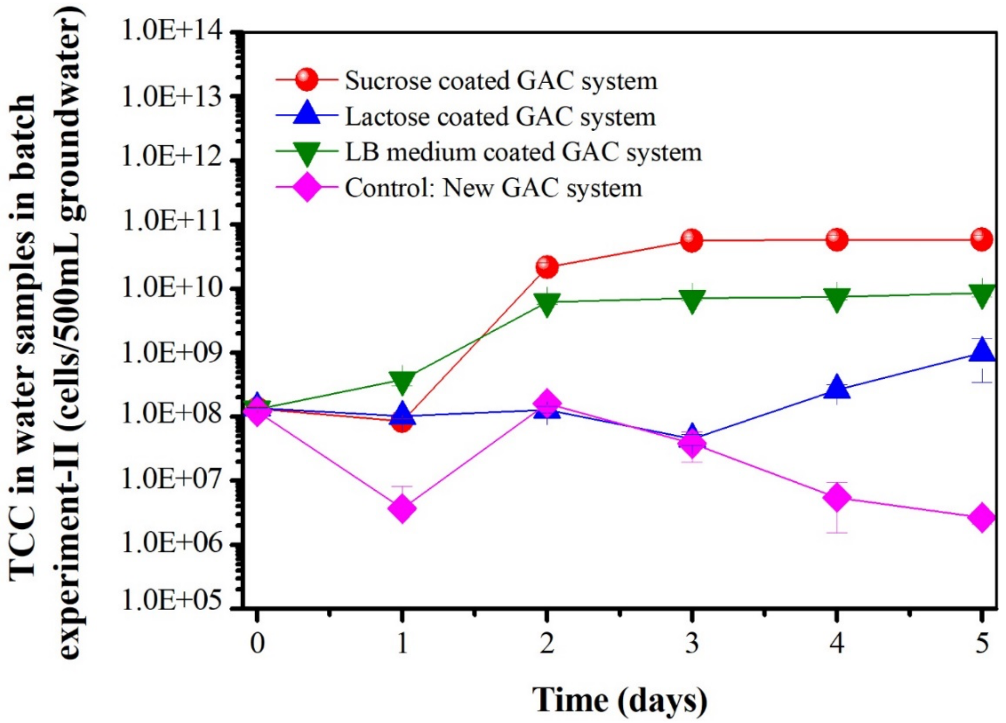

Supplementary Figure 7. (a) Intact cell concentration (ICC) in influent and effluent of different substrate coated GAC filters; (b) ICC on the GAC particles of different substrate coated GAC filters; (c) the proportion of intact bacteria accounting for the total bacteria in influent and on the GAC particles of different substrate coated GAC filters.

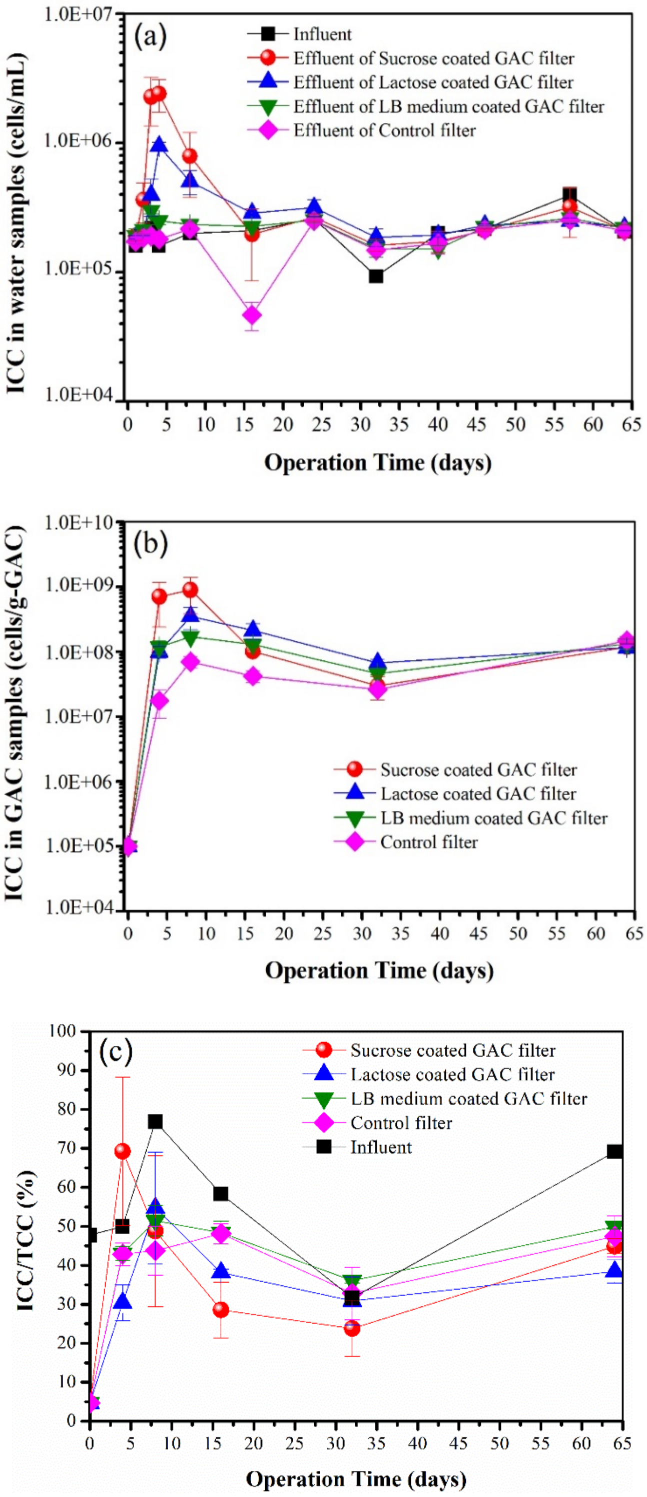

Supplementary Figure 8. Influent TOC concentrations and TOC variations of the different filters during the operation of 64 days. The positive variation represents TOC removals by the filters, and the negative variation represents increased TOC amounts from the filters.

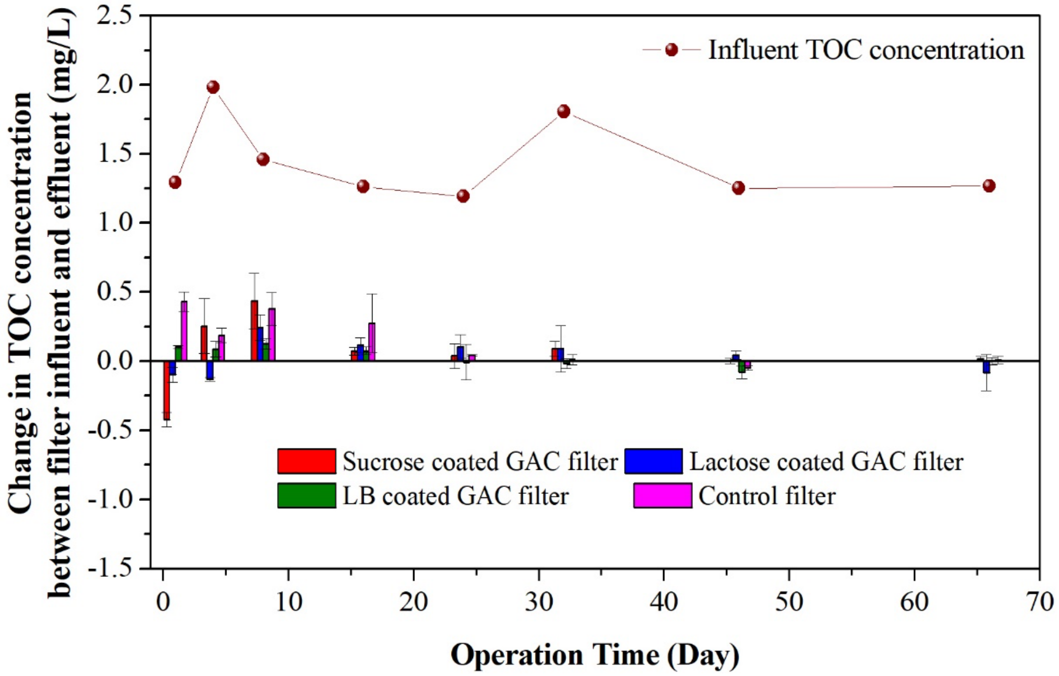

Supplementary Figure 9. Relative abundance of dominant orders in the continuous-flow GAC biofilters with the (a) sucrose, (b) lactose, (c) LB-medium, and (d) non-loaded (control) addition.

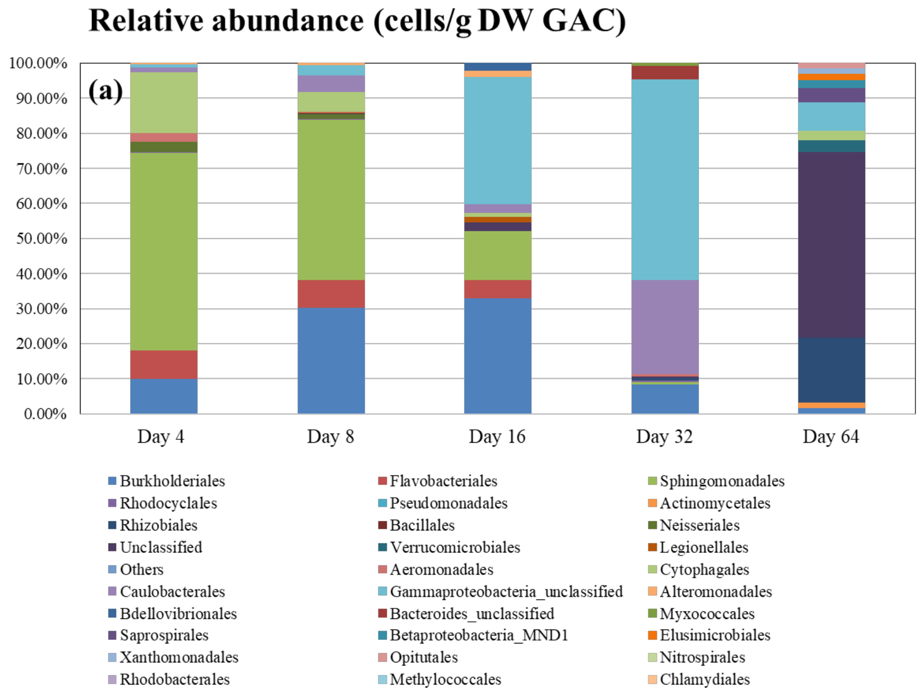

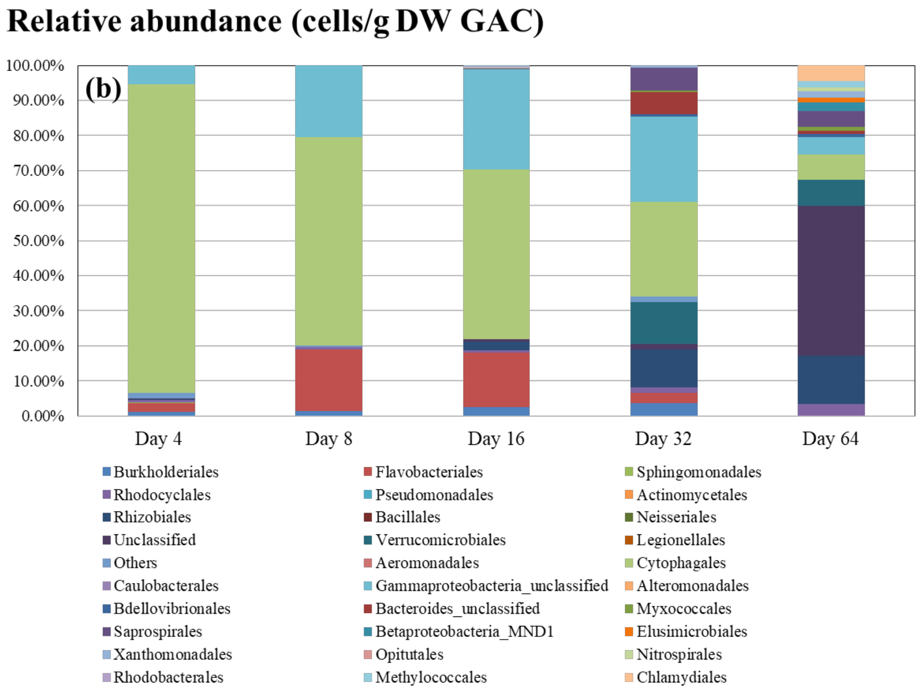

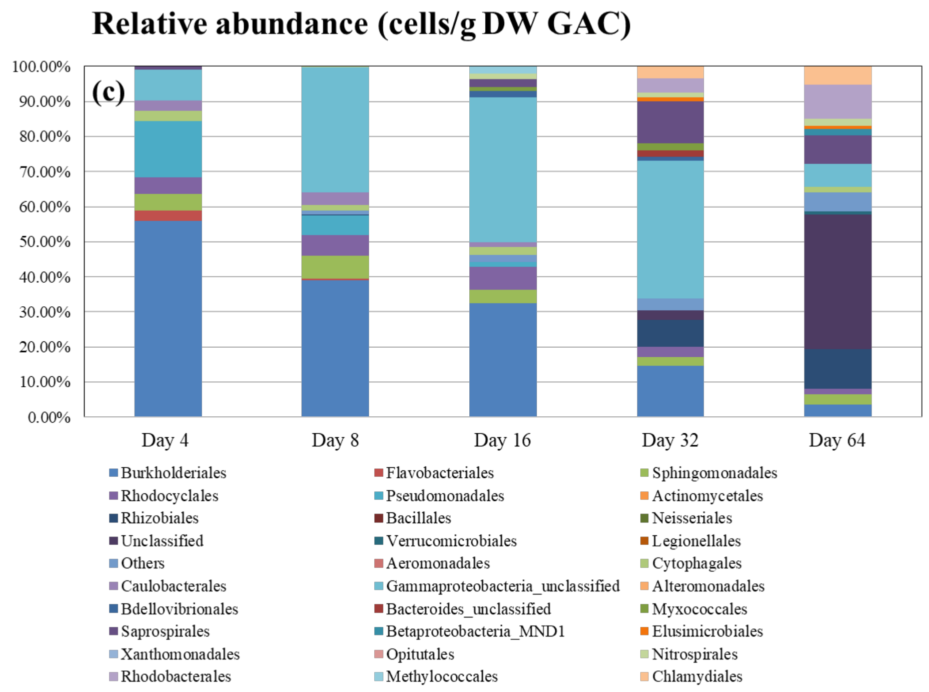
Relative abundance (cells/g DW GAC)
(c)
100.00%
90.00%
80.00%
70.00%
60.00%
50.00%
40.00%
30.00%
20.00%
10.00%
0.00%
Day 4
Day 8
Day 16
Day 32
Day 64
Burkholderiales
Flavobacteriales
Sphingomonadales
Rhodocyclales
Pseudomonadales
Actinomycetales
Rhizobiales
Bacillales
Neisseriales
Unclassified
Verrucomicrobiales
Legionellales
Others
Aeromonadales
Cytophagales
Caulobacterales
Gammaproteobacteria_unclassified
Alteromonadales
Bdellovibrionales
Bacteroides_unclassified
Myxococcales
Saprospirales
Betaproteobacteria_MND1
Elusimicrobiales
Xanthomonadales
Opitutales
Nitrospirales
Rhodobacterales
Methylococcales
Chlamydiales

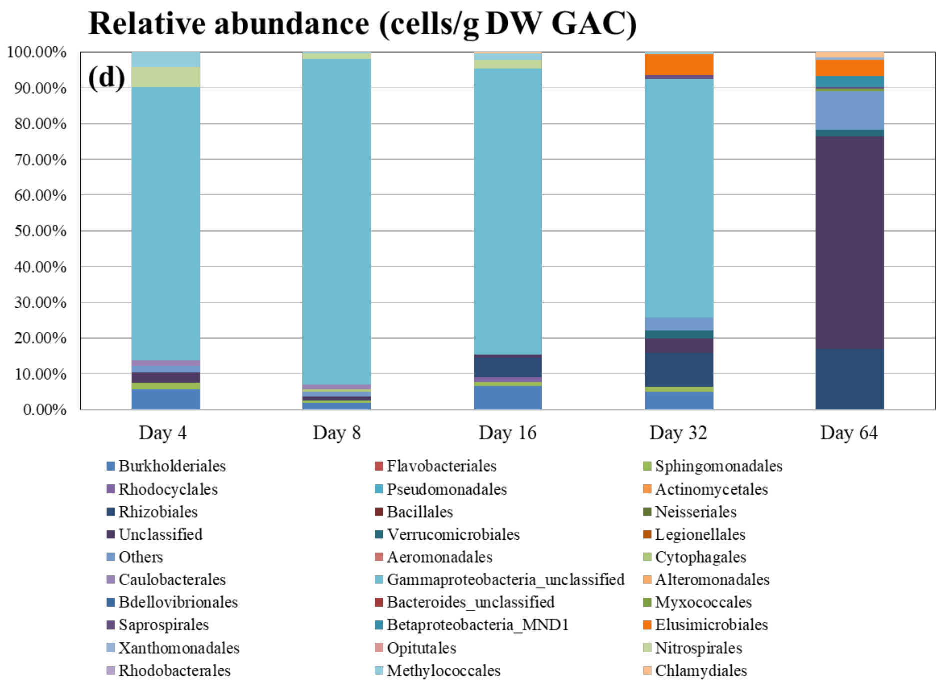
Relative abundance (cells/g DW GAC)
(d)
100.00%
90.00%
80.00%
70.00%
60.00%
50.00%
40.00%
30.00%
20.00%
10.00%
0.00%
Day 4
Day 8
Day 16
Day 32
Day 64
Burkholderiales
Flavobacteriales
Sphingomonadales
Rhodocyclales
Pseudomonadales
Actinomycetales
Rhizobiales
Bacillales
Neisseriales
Unclassified
Verrucomicrobiales
Legionellales
Others
Aeromonadales
Cytophagales
Caulobacterales
Gammaproteobacteria_unclassified
Alteromonadales
Bdellovibrionales
Bacteroides_unclassified
Myxococcales
Saprospirales
Betaproteobacteria_MND1
Elusimicrobiales
Xanthomonadales
Opitutales
Nitrospirales
Rhodobacterales
Methylococcales
Chlamydiales

Supplementary Figure 10. NMDS: Non-metric multi-dimensional scaling representation of Bray-Curtis dissimilarities between samples. NMDS was performed with phyloseq. Outline color represents the sample type: influent water, and type of carbon added for GAC media.

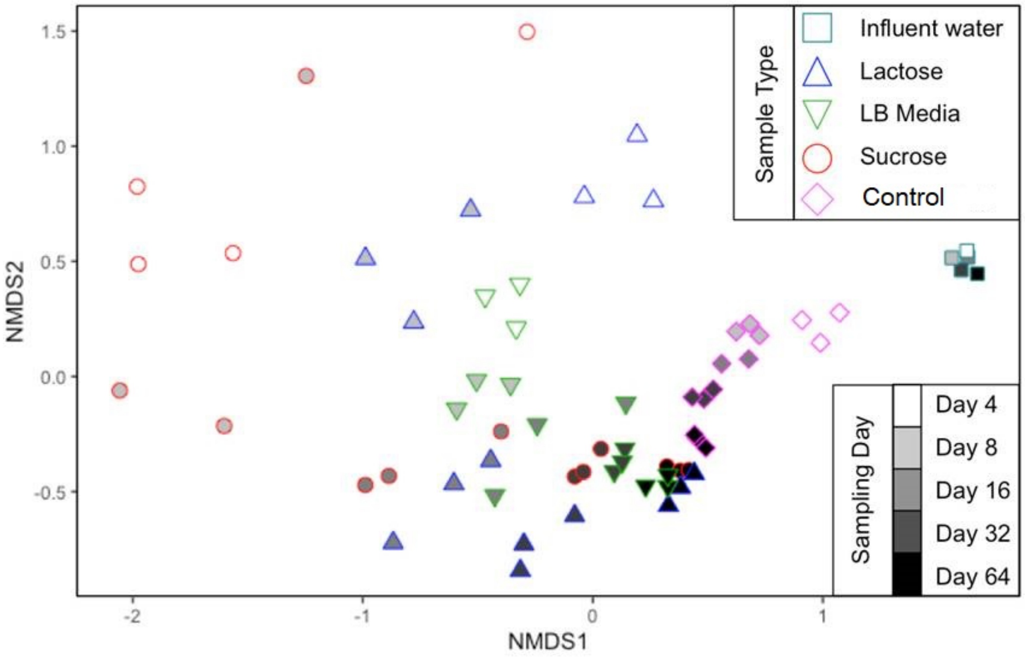

Supplementary Figure 11. Absolute abundance in influent of batch experiments and continuous experiments.

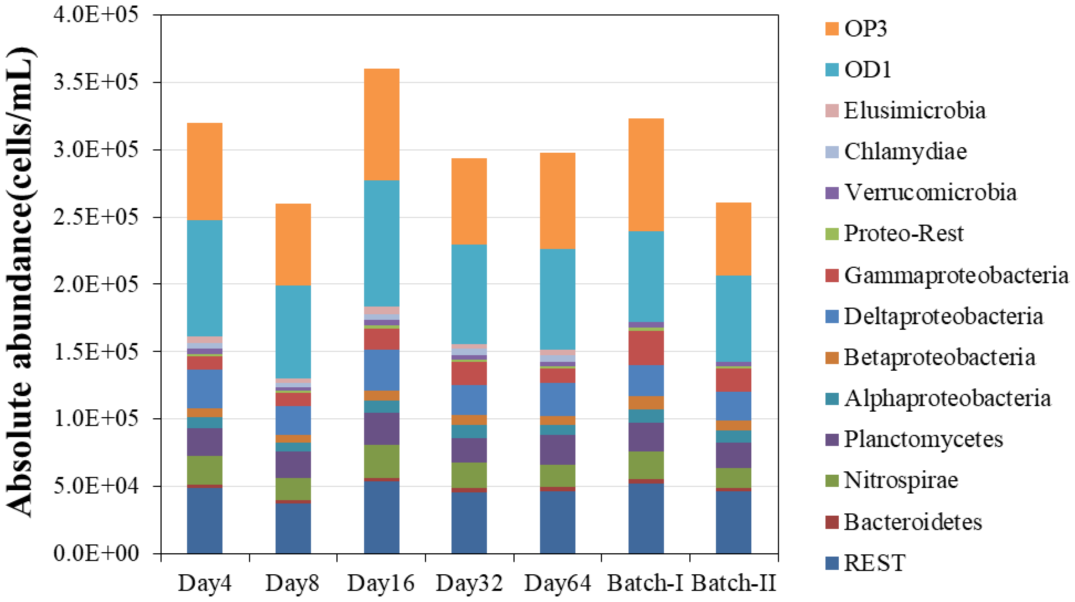

1.3 Supplementary Table

Supplementary Table 1. Primers used for 16S Amplicon PCR.

Assay	Name	Sequence
Amplicon PCR for sequencing	Bakt_341F (S-D-Bact-0341-b-S-17) // 341 F	CCTACGGGNGGCWGCAG
	Bakt_805R(S-D-Bact -0785-a-A-21) // 785 R	GACTACHVGGGTATCTAATCC
	Nextera adapters tail before forward	TCG-TCG-GCA-GCG-TCA-GAT-GTG-TAT-AAG-AGA-CAG-GAG
	Nextera adapters tail before reverse	GTC-TCG-TGG-GCT-CGG-AGA-TGT-GTA-TAA-GAG-ACA-GAG

Supplementary Table 2. Records of Index PCR.

NO.	Sample name	Index 1	Index 2
1	day4-A1	**N701**	**S513**
2	day4-A2	**N702**	**S513**
3	day4-A3	**N703**	**S513**
4	day4-B1	**N704**	**S513**
5	day4-B2	**N705**	**S513**
6	day4-B3	**N706**	**S513**
7	day4-C1	**N707**	**S513**
8	day4-C2	**N710**	**S513**
9	day4-C3	**N711**	**S513**
10	day4-D1	**N712**	**S513**
11	day4-D2	**N714**	**S513**
12	day4-D3	**N715**	**S513**
13	Day8-A1	**N701**	**S515**
14	Day8-A2	**N702**	**S515**
15	Day8-A3	**N703**	**S515**
16	Day8-B1	**N704**	**S515**

17	Day8-B2	**N705**	**S515**
18	Day8-B3	**N706**	**S515**
19	Day8-C1	**N707**	**S515**
20	Day8-C2	**N710**	**S515**
21	Day8-C3	**N711**	**S515**
22	Day8-D1	**N712**	**S515**
23	Day8-D2	**N714**	**S515**
24	Day8-D3	**N715**	**S515**
25	Day16-A1	**N701**	**S516**
26	Day16-A2	**N702**	**S516**
27	Day16-A3	**N703**	**S516**
28	Day16-B1	**N704**	**S516**
29	Day16-B2	**N705**	**S516**
30	Day16-B3	**N706**	**S516**
31	Day16-C1	**N707**	**S516**
32	Day16-C2	**N710**	**S516**
33	Day16-C3	**N711**	**S516**
34	Day16-D1	**N712**	**S516**
35	Day16-D2	**N714**	**S516**
36	Day16-D3	**N715**	**S516**
37	Day32-A1	**N701**	**S517**
38	Day32-A2	**N702**	**S517**
39	Day32-A3	**N703**	**S517**
40	Day32-B1	**N704**	**S517**
41	Day32-B2	**N705**	**S517**
42	Day32-B3	**N706**	**S517**
43	Day32-C1	**N707**	**S517**
44	Day32-C2	**N710**	**S517**
45	Day32-C3	**N711**	**S517**
46	Day32-D1	**N712**	**S517**
47	Day32-D2	**N714**	**S517**
48	Day32-D3	**N715**	**S517**
49	Day64-A1	**N701**	**S518**
50	Day64-A2	**N702**	**S518**
51	Day64-A3	**N703**	**S518**
52	Day64-B1	**N704**	**S518**
53	Day64-B2	**N705**	**S518**
54	Day64-B3	**N706**	**S518**

55	Day64-C1	**N707**	**S518**
56	Day64-C2	**N710**	**S518**
57	Day64-C3	**N711**	**S518**
58	Day64-D1	**N712**	**S518**
59	Day64-D2	**N714**	**S518**
60	Day64-D3	**N715**	**S518**
61	I-Sucrose-1	**N701**	**S520**
62	I-Sucrose-2	**N702**	**S520**
63	I-Sucrose-3	**N703**	**S520**
64	I-lactose-1	**N704**	**S520**
65	I-lactose-2	**N705**	**S520**
66	I-lactose-3	**N706**	**S520**
67	I-LB-1	**N707**	**S520**
68	I-LB-2	**N710**	**S520**
69	I-LB-3	**N711**	**S520**
70	I-Control-1	**N712**	**S520**
71	I-Control-2	**N714**	**S520**
72	I-Control-3	**N715**	**S520**
73	II-Sucrose-1	**N701**	**S521**
74	II-Sucrose-2	**N702**	**S521**
75	II-Sucrose-3	**N703**	**S521**
76	II-lactose-1	**N704**	**S521**
77	II-lactose-2	**N705**	**S521**
78	II-lactose-3	**N706**	**S521**
79	II-LB-1	**N707**	**S521**
80	II-LB-2	**N710**	**S521**
81	II-LB-3	**N711**	**S521**
82	II-Control-1	**N712**	**S521**
83	II-Control-2	**N714**	**S521**
84	II-Control-3	**N715**	**S521**
85	day4-In	**N701**	**S522**
86	day8-In	**N702**	**S522**
87	day16-In	**N703**	**S522**
88	day32-In	**N704**	**S522**
89	day64-In	**N705**	**S522**
90	Blank-1	**N706**	**S522**
91	II-In	**N707**	**S522**
92	I-In	**N710**	**S522**

93	day4-A2-2	**N711**	**S522**
94	II-lactose-3-2	**N712**	**S522**
95	II-LB-2-2	**N714**	**S522**
96	Blank-2	**N715**	**S522**
